# Differences in health behaviors and health outcomes among non-Hispanic Whites and Arab Americans in a population-based survey in California

**DOI:** 10.1186/s12889-019-7233-z

**Published:** 2019-07-08

**Authors:** Nadia N. Abuelezam, Abdulrahman M. El-Sayed, Sandro Galea

**Affiliations:** 1 Boston College, William F. Connell School of Nursing, 140 Commonwealth Avenue, Maloney Hall 352, Chestnut Hill, MA 02467 USA; 2 University of Michigan Medical School, Ann Arbor, MI USA; 3 School of Public Health, Boston University, Boston, MA USA

**Keywords:** Arab American, Chronic disease, Health behaviors, Minority health, Health disparities, Immigrant health

## Abstract

**Background:**

Few population-based studies of Arab American health behaviors and outcomes exist outside of Michigan. We aimed to provide prevalence estimates of health behaviors and outcomes for Arab Americans and compare them to non-Hispanic Whites in California.

**Methods:**

We used data from the 2003–2016 California Health Interview Surveys. We determined Arab American ethnicity using an algorithm that considered place of birth of the respondent or parent and use of Arabic language at home. Survey-weighted frequencies, chi-squared statistics, and logistic regression analyses were used to compare Arab Americans and non-Hispanic Whites on socioeconomic indicators, health behaviors and health outcomes. Multivariable models were adjusted for age, education level, and insurance status.

**Results:**

Arab Americans had higher prevalence of no insurance, living below the federal poverty level, and home ownership than non-Hispanic Whites despite high levels of education and low unemployment prevalence. Arab Americans had reduced odds of alcohol consumption (OR: 0.33, 95% CI: 0.24, 0.45), binge drinking (OR: 0.28, 95% CI: 0.19, 0.40), and suicidal ideation (OR: 0.41, 0.25, 0.66) when compared to non-Hispanic Whites in multivariable models. Arab Americans had decreased odds of hypertension (OR: 0.64, 95% CI: 0.50, 0.83) and increased odds of diabetes (OR: 2.03, 95% CI: 1.23, 3.34) when compared to non-Hispanic Whites in multivariable models.

**Conclusions:**

Arab Americans in California participate in less risky health behaviors and have better health outcomes than non-Hispanic Whites, except with regards to diabetes. Future work aiming to understand the health of Arab Americans should allow for self-identification and less reliance on country of origin and language use at home for sample selection.

## Background

Arab Americans are an understudied minority population in the United States (US) for whom health research is slowly and steadily increasing [1]. It is estimated that there are approximately 3.7 million Arab Americans in the United States [2]. California has the largest number of Arab Americans while Michigan has the highest proportion of Arab Americans in the population [2]. The majority of studies examining Arab American health in the US do so in Michigan in the context of an ethnic enclave (Dearborn, Michigan) due to ease of identification and recruitment [1]. There is reason to believe, however, that Arab Americans living in ethnic enclaves differ from those living outside of them [3]. Very few population-based studies [4–7] allow the examination of the potential bias that may occur when categorizing Arab Americans as non-Hispanic Whites in health studies outside of Michigan due to difficulties in identifying Arab Americans.

Arab Americans lack an ethnic/racial identifier due to Office of Management and Budget standards created in 1997 [8]. Persons who classify themselves as Arab American are told to self-identify as White, and in some cases, like in the case of the Census, are recoded from Other to White based on an indication of origin from the Middle East and North Africa [9]. Investigators interested in Arab American health are therefore unable to isolate Arab populations despite evidence that Arab Americans have different health behaviors, different levels of healthcare access, and experience more cultural stigma that affects health when compared to the general non-Hispanic White population [1, 10]. Additionally, the social pressures this group faces, including an increased risk of hate crimes [11] and other instances of racism, influence health in this population.

We aimed to better understand how the health of Arab Americans differs from non-Hispanic Whites in California by: 1) estimating the prevalence of various health outcomes and behaviors among Arab Americans and 2) examining the association between race/ethnicity and these health outcomes and behaviors while adjusting for confounders.

## Methods

### California health interview survey

We used the California Health Interview Survey (CHIS), the largest state health survey conducted in the US. Since 2001, the CHIS has collected data on demographics, health, and healthcare needs of 20,000–40,000 Californians every year [12]. The CHIS uses a dual-frame landline and cell phone random-digit-dialing sample of the non-institutionalized California household population. The two-stage, geographically stratified design is used to produce a representative sample of the state [12]. From 2001 to 2009 CHIS data was collected every 2 years while from 2011 to 2017 the survey has been conducted annually. For all sampled households, one randomly selected adult is asked survey questions. Survey questions were not consistently asked in all years, leading to varying survey lengths, and missing data is imputed using rigorously developed methodologies [13]. Interviews are conducted with a computer-assisted telephone interviewing system and on average took 41 min to complete. The response rates have declined over time from 60.0% (landlines) [14] in 2003 to 41.5% (landlines) and 47.9% (cell phones) in 2015/2016 [12]. We used data from the 2003–2016 CHIS survey cycles in this study. The survey asks respondents to answer questions about their sociodemographic characteristics, their health behaviors, health conditions, and knowledge about health.

### Study population

Using the CHIS Adult Questionnaire (respondents are 18 years or older), we isolated Arab Americans through their open-ended responses to three questions: “*In what country were you born?*”, “*In what country was your mother born?*”, or “*In what country was your father born?*” If a respondent indicated one of 22 Arab League countries, they were coded as an Arab American respondent. Additionally, we were able to isolate other Arab Americans through the question: “*What languages do you speak at home?*” where Arabic language spoken at home was coded as an Arab American respondent.

Non-Hispanic White respondents were identified through their responses to two sequential questions: “*Are you Latino or Hispanic?*” and “*Please tell me which one or more of the following you would use to describe yourself*” with the option of “No” indicated for Hispanic or Latino origin and “White” selected for racial identification by the respondent. Any respondent who answered non-Hispanic White and was determined to not be of Arab descent was included in this category.

### Demographic and socioeconomic status variables

We used the following demographic variables that were available for all survey years: sex, age (18–29, 30–49, 50+ years of age), marital status (married vs. not married), nativity (born inside vs. outside US), citizenship status (citizen vs. non-citizen), and length of time lived in the US (less than vs. greater than 5 years). Socioeconomic variables examined, available for all survey years, include unemployment status (employed vs. unemployed), educational attainment (high school or less vs. some college or more), living below the federal poverty level (0–99%), health insurance status (uninsured vs. insured), and home ownership (own vs. rent). Variables were dichotomized, where possible, to simplify the analysis and to account for the largest differences between categories.

### Health behaviors

We aimed to examine health behaviors that were relevant for the health and wellbeing of the general adult population in California and that were included as questions on CHIS, excluding screening tests. We were specifically interested in health behaviors that played a role in preventing chronic disease and death in adulthood.

Health behaviors examined include self-reported flu vaccination in the past year (flu vaccine vs. no flu vaccine, 2003–2016), drinking soda 5+ times per week (5+ times vs. < 5 times, 2005–2016), never smoking (never smoking vs. current smoking/quit smoking, 2003–2016), having alcohol in the past year (alcohol vs. no alcohol, 2007–2016), binge drinking in the past year (5 or more drinks in one sitting vs. < 5 drinks/none, 2007–2016), having 2+ sexual partners in the past year (2+ partners vs. 0–1 partner, 2003–2016), reporting an ER visit in the past year (ER visit vs. none, 2005–2016), and ever having contemplated committing suicide (contemplated suicide vs. not contemplated, 2009–2016).

### Health outcomes

We examined health outcomes, available across all survey years, that have been explored in other population-based studies in the Arab American health literature [15]. Health outcomes examined include self-rated poor/fair health (fair/poor vs. good/very good/excellent), diabetes (pre-diabetes and diabetes vs. none), high blood pressure (pre- hypertension and hypertension vs. none), heart disease (heart disease vs. none), and overweight or obesity (overweight/obese vs. normal weight).

### Analysis

All analyses accounted for sampling and replicate weights to adjust for the complex survey sampling design using the jack knife repeated replication method. Population-weighted frequencies and proportions were used in all analyses. Chi-square tests were used to compare Arab Americans to non-Hispanic Whites by demographic, socioeconomic status, health behavior, and health outcome indicators. Survey weighted logistic regression was performed for each dichotomized health behavior and health outcome of interest. Unadjusted univariate analyses were run with an indicator for heritage from an Arabic speaking country. Adjusted analyses included dichotomous age (< 40/40+), education (high school or less/college or more), and insurance status (insured/uninsured) in the multivariable models. Age was dichotomized in this manner to adjust for the increased risk for chronic disease among individuals age 40 and over [16].

## Results

A total of 1359 Arab Americans and 192,868 non-Hispanic Whites were identified from the CHIS between 2003 and 2016. More male Arab Americans were identified than non-Hispanic Whites (54.9 vs. 49.0%, *p* < 0.05), and the age distribution of the two groups differed with more Arab Americans aged 30–49 (42.2 vs. 33.8% among non-Hispanic Whites, *p* < 0.001) and more non-Hispanic Whites aged 50 or over (49.9 vs. 25.5% among Arab Americans, *p* < 0.001). More Arab Americans were born outside the United States (65.1 vs. 8.7%, *p* < 0.001) and fewer Arab Americans were citizens when compared to non-Hispanic Whites (84.9 vs. 90.6%, *p* < 0.05). Table 1 compares demographic, socioeconomic, and health related factors between Arab Americans and non-Hispanic Whites.

**Table 1:** Characteristics of Arab American and non-Hispanic White participants, N (population-weighted %), of the California Health Interview Survey between 2003 and 2016 used for this analysis. Participants are compared on demographic, socioeconomic, health behavior, and health outcome variables

	Arabic Language or Heritage (*N* = 1359), N (population-weighted %)	Non-Hispanic White (*N* = 192,865), N (population-weighted %)
*Demographic*		
Male	661 (54.9)	78,728 (49.0)
Age		
18–29	265 (32.3)	12,916 (16.4)
30–49	482 (42.2)	45,104 (33.8)
50+	612 (25.5)	133,610 (49.9)
Married	788 (52.5)	97,474 (56.6)
Born outside of U.S.	885 (65.1)	14,462 (8.7)
Citizen	1200 (84.9)	177,389 (90.6)
Lived in U.S. less than 5 years	81 (8.6)	619 (0.7)
*Socioeconomic Status*		
Unemployed	533 (27.4)	91,144 (35.5)
High school or less	327 (25.2)	48,033 (28.6)
0–99% FPL	192 (13.9)	12,041 (6.4)
Uninsured (in past year)	186 (23.8)	15,977 (11.9)
Own home	822 (56.4)	143,355 (70.1)
*Health Behaviors*		
Flu vaccine (past year)	415 (25.5)	80,953 (34.5)
Drinking soda 5+ times per week	112 (12.0)	15,631 (10.4)
Never smoker	838 (62.5)	100,221 (55.2)
Alcohol in past year	517 (40.1)	98,589 (51.2)
Binge drinking in past year	110 (17.7)	30,633 (33.9)
2+ sexual partners in past year	103 (11.4)	9827 (8.4)
ER visit in past year	220 (14.5)	34,151 (17.2)
Ever thought to commit suicide	51 (3.8)	11,196 (6.3)
*Health Outcomes*		
Poor/Fair self-rated health	194 (12.5)	30,458 (13.4)
Diabetes	151 (8.6)	19,640 (7.9)
High blood pressure	346 (16.0)	72,878 (29.7)
Heart Disease	109 (3.9)	22,700 (8.2)
Overweight or obese	805 (55.2)	107,990 (55.7)

### Socioeconomic status

More Arab Americans were uninsured (23.8 vs. 11.9%, *p* < 0.001) and living at 0–99% of the federal poverty level (13.9 vs. 6.4%, *p* < 0.001) but fewer Arab Americans were unemployed (27.4 vs. 35.5%, *p* < 0.001) or had low education (25.2 vs. 28.6%) than non-Hispanic Whites (Table 1). Fewer Arab American owned homes when compared to non-Hispanic Whites (56.4 vs. 70.1%, *p* < 0.001).

### Health behaviors

Arab Americans had lower binge drinking (17.7 vs. 33.9%, p < 0.001) and alcohol consumption (40.1 vs. 51.2%, p < 0.001) prevalence in the past year than non-Hispanic Whites. Fewer Arab Americans received a flu vaccine (25.5 vs. 34.5%) and visited an emergency room (14.5 vs. 17.2%) in the past year than non-Hispanic Whites. Arab Americans had a higher prevalence of having two or more sexual partners in the past year (11.4 vs. 8.4%, *p* = 0.022) but lower prevalence of ever having contemplated suicide (3.7 vs. 6.3%, *p* = 0.001) than non-Hispanic Whites.

Arab Americans had significantly lower odds of reporting alcohol consumption (OR: 0.33, 95% CI: 0.24, 0.45) and binge drinking (OR: 0.28, 95% CI: 0.19, 0.40) in the past year in adjusted models than non-Hispanic Whites (Table 2). Arab Americans had significantly lower odds of ever having contemplated suicide (OR: 0.41, 95% CI: 0.25, 0.66) in adjusted models when compared to non-Hispanic Whites.

**Table 2:** Population-weighted logistic regression results comparing the odds of health behaviors and health outcomes for Arab American respondents compared to non-Hispanic White respondents in the California Health Interview Survey

	OR (95% CI), Unadjusted	OR (95% CI), Adjusted*
*Health Behaviors*		
Flu vaccine in past year	0.64 (0.49, 0.83)**	0.95 (0.69, 1.31)
Had alcohol in past year	0.41 (0.31, 0.55)**	0.33 (0.24, 0.45)**
Binge drinking in past year	0.42 (0.29, 0.61)**	0.28 (0.19, 0.40)**
2+ sexual partners in past year	1.27 (0.88, 1.82)	0.87 (0.59, 1.30)
Visited an ER in past year	0.79 (0.59, 1.06)	0.82 (0.60, 1.13)
Contemplated suicide	0.49 (0.31, 0.77)**	0.41 (0.25, 0.66)**
*Health Outcomes*		
Fair/Poor self-rated health	0.92 (0.60, 1.42)	1.18 (0.70, 1.99)
Diabetes	1.10 (0.74, 1.63)	2.03 (1.23, 3.34)**
Hypertension	0.45 (0.37, 0.54)**	0.64 (0.50, 0.83)**
Heart disease	0.45 (0.32, 0.64)**	0.60 (0.32, 1.15)
Overweight or obese	0.98 (0.79, 1.22)	1.10 (0.88, 1.37)

*Adjusted for age, education, and health insurance status

**Statistically significant (p < 0.05)

### Health outcomes

Across all health outcomes but diabetes, Arab Americans had lower adverse health prevalence than non-Hispanic Whites including significantly lower prevalence of hypertension (16.0 vs. 29.7%, *p* < 0.001) and heart disease (3.9 vs. 8.2%, p < 0.001) (Table 1).

Both unadjusted (OR: 0.45, 95% CI: 0.37, 0.54) and adjusted (OR: 0.64, 95% CI: 0.50, 0.83) logistic regression models show that Arab Americans had significantly reduced odds of self-reported hypertension when compared to non-Hispanic Whites (Table 2). Arab Americans had increased odds of self-reporting diabetes when compared to non-Hispanic Whites (OR: 2.03, 95% CI: 1.23, 3.34) in adjusted models.

## Discussion

The goals of this study were to provide estimates for the prevalence of health behaviors and outcomes among Arab Americans in California and to examine differences with non-Hispanic Whites. Overall, Arab Americans reported more positive health behaviors and health outcomes than non-Hispanic Whites in our CHIS sample. Arab Americans had decreased odds of reporting alcohol use (including binge drinking), ever contemplating suicide, and hypertension than non-Hispanic Whites. The only outcome Arab Americans had increased odds of self-reporting was diabetes.

Previous studies on alcohol use and misuse among Arab Americans in national and Michigan-based surveys have also found that Arab Americans are less likely to report alcohol use than non-Hispanic Whites [17]. Binge drinking in the past month was found to be lower among Arab Americans identified through the National Survey on Drug Use (10%) than in the Michigan Behavioral Risk Factor Survey (17%) [17]. Arab Americans immigrate to the United States from countries with low alcohol consumption rates due to the religious prohibition and social discouragement of drinking in many origin countries [18, 19]. Some theories exist as to why Arab Americans may begin using alcohol including social change and acculturation [20], trauma from origin countries [21], and the larger composition of Christian Arab Americans than Muslim Arab Americans [2], but alcohol consumption remains low in this population.

We found that Arab Americans are less likely to have ever contemplated suicide than non-Hispanic Whites. Only one other study, based in Michigan, has examined suicide among Arab Americans. That study found that Arab Americans were less likely to die of suicide than non-Arab Whites in Michigan between 1990 and 2007 [22]. Studies among ethnic minority groups suggest that Arab Americans may be at lower risk for suicide and suicidal ideation due to socially-oriented norms like communalism and strong family bonds [23]. Positive ethnic group identity and affective expression may also be important mechanisms through which the risk of suicide is reduced for these populations [23].

We found higher diabetes (8.6 vs. 7.9%), lower hypertension (16.0 vs. 29.7%), and lower heart disease prevalence (3.9 vs. 8.2%) among Arab Americans than in non-Hispanic Whites. Arab Americans had increased odds of self-reporting diabetes and lower odds of self-reporting hypertension than non-Hispanic Whites in our sample. Diabetes incidence and prevalence have been shown to be higher among immigrant populations in the US [24]. Additionally, diabetes prevalence in the Middle East is high [25] and Arab immigrants to other countries have been found to have a high burden of diabetes [26]. The pattern found in this paper, higher levels of diabetes but not hypertension or obesity, is in congruence with evidence from the National Health Interview Survey in which Middle Eastern immigrants had higher rates of diabetes but not hypertension or obesity than European immigrants to the US [27]. Some reasons why Arab Americans may be at increased risk for diabetes include changes to diet associated with acculturation [28] and the potential for lower access and under-utilization of health care for this minority population [29].

Disease prevalence in our sample differs from that examined in national health surveys and Michigan based surveys. The National Health Interview Survey found a lower diabetes prevalence among Arab Americans (4.8%, identified through place of birth) and non-Hispanic Whites (6.9%) than in our sample [15]. Hypertension prevalence was also higher in our sample than in the National Health Interview Survey for both Arab Americans (13.4%) and non-Hispanic Whites (24.5%) [15], while heart disease prevalence in our sample was lower than that reported in the Michigan Behavioral Risk Factor Survey for Arab Americans (8.8%) and non-Hispanic Whites (8.1%) [4]. The differences in chronic disease prevalence between Michigan and California is of particular concern given the reliance on Arab American data coming out of Michigan. If the health behaviors and health outcomes differ for Arab Americans in contexts outside of Michigan, this could have large ramifications for the overall knowledge of Arab American health risks nationally. Some potential reasons for the differences in chronic disease prevalence between the samples may include the methods of recruiting and isolating Arab Americans, differences in community context and acculturation, and differences in length of stay in the US of the Arab Americans sampled. In our study, individuals did not self-identify as Arab American but were isolated through the use of country of birth and language spoken at home. Studies in Michigan have traditionally allowed for self-identification of Arab American identity. These differences may result in different samples with different characteristics, including sociodemographic and immigration level variables. Additionally, differences in socioeconomic status and opportunity between California and Michigan may be driving differences in results. Although data is limited, there is some evidence to suggest that Arab Americans in California are wealthier than those living in Michigan [30] despite the poor socioeconomic indicators reported by Arab Americans in our sample. Additionally, the ethnic community context in which people live likely plays a large factor in the available nutritional and social activities that may influence these outcomes. The concentration of Arab Americans in Michigan is higher than that in California and may therefore drive ethnic community context [31]. Finally, some work has shown that length of residence in the United States is directly correlated with obesity and chronic disease risk [32] and length of stay in the US may differ from state to state.

There are some limitations to our analysis. First, the use of random digit dialing for recruitment may not be yielding a representative sample of Arab Americans. The Arab American Institute estimates approximately 817,455 Arab Americans living in California (a state with an adult population of 34.4 million). We estimate therefore that approximately 2% of the total adult Californian population is Arab American. Since the CHIS enrollment is estimated at 20,000 Californians every year, we might expect that 400 Arab Americans should be identified each year through the survey. Our sample shows 80–150 Arab Americans are being recruited annually suggesting under-recruitment of this population or an inability to properly identify Arab Americans using the questions on birth place and language. Second, all outcomes are self-reported by the participant and are not verified with medical records potentially leading to reporting bias due to stigma and shame associated with particular health behaviors and outcomes [17]. Third, due to the lack of recruitment of a large number of Arab Americans, we were underpowered to detect differences in particular health outcomes and health behaviors. Finally, the group of Arab Americans isolated from CHIS is heterogeneous and this heterogeneity may mask disease risk patterns for this population. Specifically, the inclusion of Arabic speaking individuals from North Africa, who may self-identify as Black on standard race surveys, may in fact have different social experiences and exposures that may put them at differential risk for various health outcomes.

## Conclusions

Despite these limitations, our study reports on one of the largest rigorously conducted population-based samples of Arab Americans from California and provides one of the first looks at differences between Arab Americans and non-Hispanic Whites in California on a number of health behaviors and outcomes. We found Arab Americans participated in less risky health behaviors and had better health outcomes, except with regard to diabetes. Future work in California should allow for self-identification of Arab American ethnicity in order to properly understand the health needs of this minority population. Understanding the differences in demographics, socioeconomic status, health behaviors, and health outcomes between Arab Americans and non-Hispanic Whites in California will help public health officials and clinicians better target health messaging and health promotion to Arab Americans, an important minority subgroup.

## Data Availability

The data that support the findings of this study are available from the UCLA Center for Health Policy Research but restrictions apply to the availability of these data, which were used under license for the current study, and so are not publicly available. Data are however available from the authors upon reasonable request and with permission of UCLA Center for Health Policy Research.

## Abbreviations

CHIS: California Health Interview Survey
US: United States
